# ARE INTRINSIC CAPACITY AND MULTIMORBIDITY ASSOCIATED TO FRIED’S FRAILTY REVERSIBILITY?

**DOI:** 10.1093/geroni/igac059.1286

**Published:** 2022-12-20

**Authors:** Emmanuel Gonzalez Bautista, Aarón Salinas Rodríguez, Ana Rivera Almaraz, Betty Soledad Manrique Espinoza

**Affiliations:** Institute of Aging. Toulouse University Hospital (CHU Toulouse)., Toulouse, Midi-Pyrenees, France; National Institute of Public Health, Cuernavaca, Morelos, Mexico; National Institute of Public Health, Cuernavaca, Morelos, Mexico; National Institute of Public Health, Cuernavaca, Morelos, Mexico

## Abstract

Our objective was to characterize older adults with reversible frailty in their baseline intrinsic capacity and multimorbidity status. MethodsWe used data from the most recent waves of the SAGE Mexico study (3 and 4), representative of older adults at a national level. Study n=749.We objectively measured gait speed and grip strength based on Fried's frailty criteria. Weight loss exhaustion was self-reported and physical activity (using the IPAQ).Reversible frailty was defined as going from frailty to pre-frail or robust and from pre-frail to robust. Worsening and stable frailty were coded similarly. Intrinsic capacity was measured using a summary index (0-100) based on five domains: cognition, locomotion, sensory, nutrition, and psychological. Multimorbidity was coded yes/no if the participant self-reported two or more chronic conditions. We compared the odds of being in the reverse group versus not in it according to baseline intrinsic capacity score and multimorbidity status, adjusting for age, sex, rural/urban, and wealth. ResultsReversible frailty=33% of those pre-frail or robust at baseline. Intrinsic capacity was higher in the reverse group than in those with worsening frailty but not significantly higher than those with stable frailty.Having multimorbidity significantly decreased the chances of frailty reversibility, adjusting for covariates. ConclusionsAs an expression of lifecourse damage to the physiological reserve, multimorbidity limits the chances of reversible frailty in Mexican older adults. High levels of intrinsic capacity did not characterize frailty reversibility in our study. Yet, low intrinsic capacity levels are a marker for older adults prone to frailty worsening.

